# Histamine H_3_ Receptor Integrates Peripheral Inflammatory Signals in the Neurogenic Control of Immune Responses and Autoimmune Disease Susceptibility

**DOI:** 10.1371/journal.pone.0062743

**Published:** 2013-07-22

**Authors:** Dimitry N. Krementsov, Emma H. Wall, Rebecca A. Martin, Meenakumari Subramanian, Rajkumar Noubade, Roxana Del Rio, Gary M. Mawe, Jeffrey P. Bond, Matthew E. Poynter, Elizabeth P. Blankenhorn, Cory Teuscher

**Affiliations:** 1 Department of Medicine, Immunobiology Program, University of Vermont, Burlington, Vermont, United States of America; 2 Department of Microbiology and Immunology, Drexel University College of Medicine, Philadelphia, Pennsylvania, United States of America; 3 Department of Pathology, University of Vermont, Burlington, Vermont, United States of America; University of Muenster, Germany

## Abstract

Histamine H_3_ receptor (*Hrh3*/H_3_R) is primarily expressed by neurons in the central nervous system (CNS) where it functions as a presynaptic inhibitory autoreceptor and heteroreceptor. Previously, we identified an H_3_R-mediated central component in susceptibility to experimental allergic encephalomyelitis (EAE), the principal autoimmune model of multiple sclerosis (MS), related to neurogenic control of blood brain barrier permeability and peripheral T cell effector responses. Furthermore, we identified *Hrh3* as a positional candidate for the EAE susceptibility locus *Eae8*. Here, we characterize *Hrh3* polymorphisms between EAE-susceptible and resistant SJL and B10.S mice, respectively, and show that *Hrh3* isoform expression in the CNS is differentially regulated by acute peripheral inflammatory stimuli in an allele-specific fashion. Next, we show that *Hrh3* is not expressed in any subpopulations of the immune compartment, and that secondary lymphoid tissue is anatomically poised to be regulated by central H_3_R signaling. Accordingly, using transcriptome analysis, we show that, inflammatory stimuli elicit unique transcriptional profiles in the lymph nodes of H_3_RKO mice compared to WT mice, which is indicative of negative regulation of peripheral immune responses by central H_3_R signaling. These results further support a functional link between the neurogenic control of T cell responses and susceptibility to CNS autoimmune disease coincident with acute and/or chronic peripheral inflammation. Pharmacological targeting of H_3_R may therefore be useful in preventing the development and formation of new lesions in MS, thereby limiting disease progression.

## Introduction

Multiple sclerosis (MS), a chronic inflammatory disease of the central nervous system (CNS), is the most common disabling neurologic disease of young adults and adolescents affecting ∼350,000 individuals in the United States and more than 1 million individuals worldwide [1]. The etiopathogenesis of MS is largely unknown; however, it involves both genetic and environmental factors [2], [3], [4]. The spectrum of clinical courses in MS is diverse and includes relapsing/remitting (R/R), primary progressive, secondary progressive, and progressive relapsing MS [5]. Additional subtypes based on severity include benign [6] and malignant MS [7], [8]. The pathologic lesions that best correlate with acute clinical exacerbations of disease feature foci of inflammation associated with active myelin degradation and phagocytosis and partial axonal preservation. Axonal injury and loss, however, occur to varying degrees in all lesions and in normal-appearing white and grey matter, and axon loss is a major correlate for permanent clinical deficits. Structurally, MS lesions show characteristic features which include demyelination, loss of oligodendrocytes, preferential destruction of thin caliber axons, impaired remyelination and astrocytic gliosis [9].

Research into the mechanisms underlying neuroinflammatory reactions in MS is largely driven by two hypotheses [10]. The immune-initiated hypothesis contends that autoreactive T cells generated in the periphery gain entry to the CNS where they elicit an inflammatory cascade that results in injury to previously normal neural tissues. In contrast, the neural-initiated disease hypothesis posits that events within the CNS initiate the process and that autoimmune responses are secondary. Previously, in the course of our studies examining the role of histamine and histamine receptors in experimental autoimmune encephalomyelitis (EAE) – often used to model aspects of these essentially conflicting hypotheses – we identified histamine H_3_ receptor (*Hrh3*/H_3_R) as a gene that potentially unites these opposing theories functionally [11].

H_3_R is expressed presynaptically where it is an inhibitory autoreceptor (inhibits release of HA from histaminergic neurons) and heteroreceptor (inhibits release of other neurotransmitters such as acetylcholine, noradrenaline, dopamine, 5-HT, GABA, and glutamate from non-histaminergic neurons) [12]. Absence of presynaptic inhibition results in failure to limit neurotransmitter release, increased postsynaptic activity, and neurotransmitter spillover [13]. Our studies revealed the existence of an H_3_R-mediated central component in susceptibility to EAE related to neurogenic control of blood brain barrier (BBB) permeability and expression of cytokines and chemokines, and their receptors, by peripheral T cells. Subsequently, H_3_R was shown to similarly regulate neuroinflammation in cerebral malaria, with H_3_R-deficiency correlating with increased BBB permeability and altered T cell phenotypes [14]. Moreover, we identified *Hrh3*, which under normal physiologic conditions plays a role in regulating body weight [15], as a positional candidate gene for *Eae8*, a quantitative trait locus (QTL) controlling EAE susceptibility and associated weight loss [16], [17], [18].

In this study, we provide functional characterization of a G293D polymorphism in the third intracellular domain of H_3_R associated with G_i/o_ and beta-arrestin coupling to second messenger signaling pathways [19], [20], [21] that distinguishes EAE-susceptible SJL mice and EAE-resistant B10.S mice. We also demonstrate allele-specific differential expression of *Hrh3* isoforms in the CNS in response to peripheral inflammatory stimuli, i.e., adjuvants used to elicit disease. Using a transcriptomics approach, we further show that the absence of H_3_R signaling in the CNS significantly alters early responses to such stimuli at the level of the lymph node (LN). Taken together, our results provide additional support for *Hrh3* as a gene central to a neural reflex [22], [23], [24] controlling peripheral immune responses and EAE susceptibility, and as a positional candidate gene for *Eae8.* Importantly, our findings provide a functional framework uniting the immune- and neural-initiated models of MS pathogenesis, and provide insight into the mechanisms whereby gene-by-environmental stimuli may influence the long term progression and spectrum of clinical disease courses seen in MS [25].

## Results and Discussion

### Characterization of Hrh3 Polymorphism Distinguishing EAE-susceptible SJL and EAE-resistant B10.S Mice

**Figure 1:**
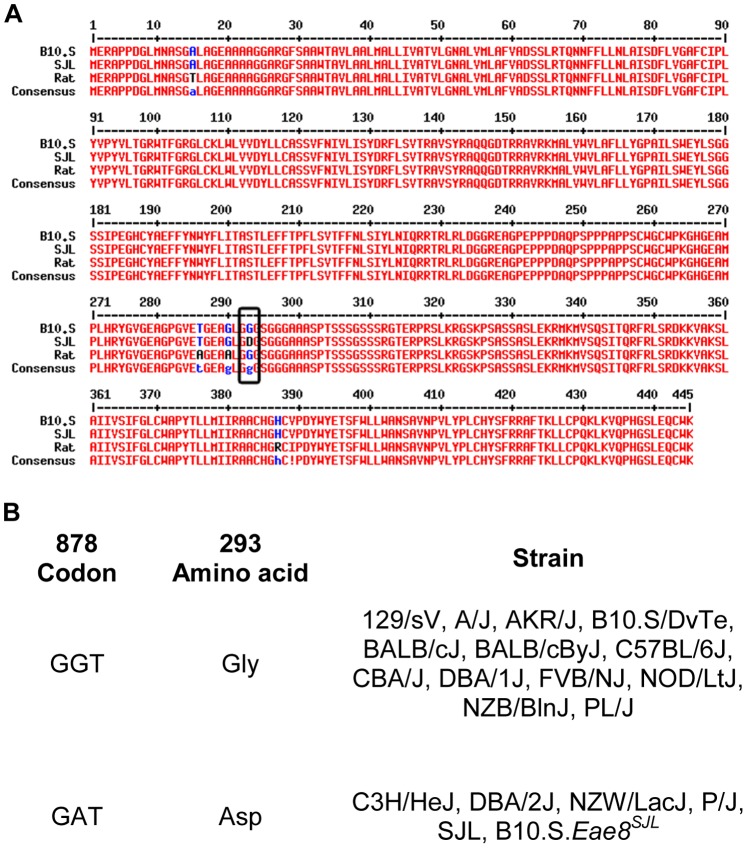
Murine *Hrh3* demonstrates a single nucleotide polymorphism. (**A**) Sequence alignment of *Hrh3* alleles from B10.S and SJL mice, and rat. *Hrh3* cDNAs from the B10.S and SJL mice were amplified, subcloned, and sequenced, as described in the Materials and Methods. (**B**) The strain distribution of the SNP at position 293 in *Hrh3*. The presence of the SNP in the indicated strains of mice was determined using restriction typing, as described in the Materials and Methods.

Previously, using B10.S.SJL-*Eae8* congenic mice, we identified *Hrh3* as a positional candidate gene for *Eae8*, a QTL controlling EAE susceptibility and associated weight loss [16], [17], [18]. Given that EAE-susceptible mouse strains experience weight loss with the onset of EAE [26] and that H_3_RKO mice manifest changes in weight and energy expenditure [27], we hypothesized that an *Hrh3* polymorphism distinguishing EAE-resistant B10.S and EAE-susceptible SJL mice may underlie *Eae8*. As a first test of this hypothesis, we undertook cDNA sequencing of the two alleles to identify coding region variants. A single missense mutation at position 878 (G to A) leading to a change from glycine to aspartic acid at residue 293 (G293D in SJL) was identified (**Fig. 1**). An examination of 18 different inbred strains of mice using restriction fragment length polymorphism (RFLP) analysis confirmed the existence of two alleles segregating among the various inbred strains **(** **Fig. 1** **)**.

The G293D substitution resides within the third intracellular (IC) loop which couples H_3_R to G_i/o_ and beta-arrestin second messenger signaling pathways [19], [20], [21]. The G293D substitution in H_3_R is analogous to the amino acid substitutions recently identified within the third IC domain of H_1_R underlying *Bphs*, a shared immunopathology disease gene controlling *Bordetella pertussis*-induced sensitivity to histamine, EAE, and autoimmune orchitis [28], [29], [30]. Importantly, a A280V missense mutation within the third IC loop of the human H_3_R gene has been reported in multiple system atrophy (Shy-Drager Syndrome), a rare neurodegenerative disease [31] and as a risk factor for migraine [32].

**Figure 2:**
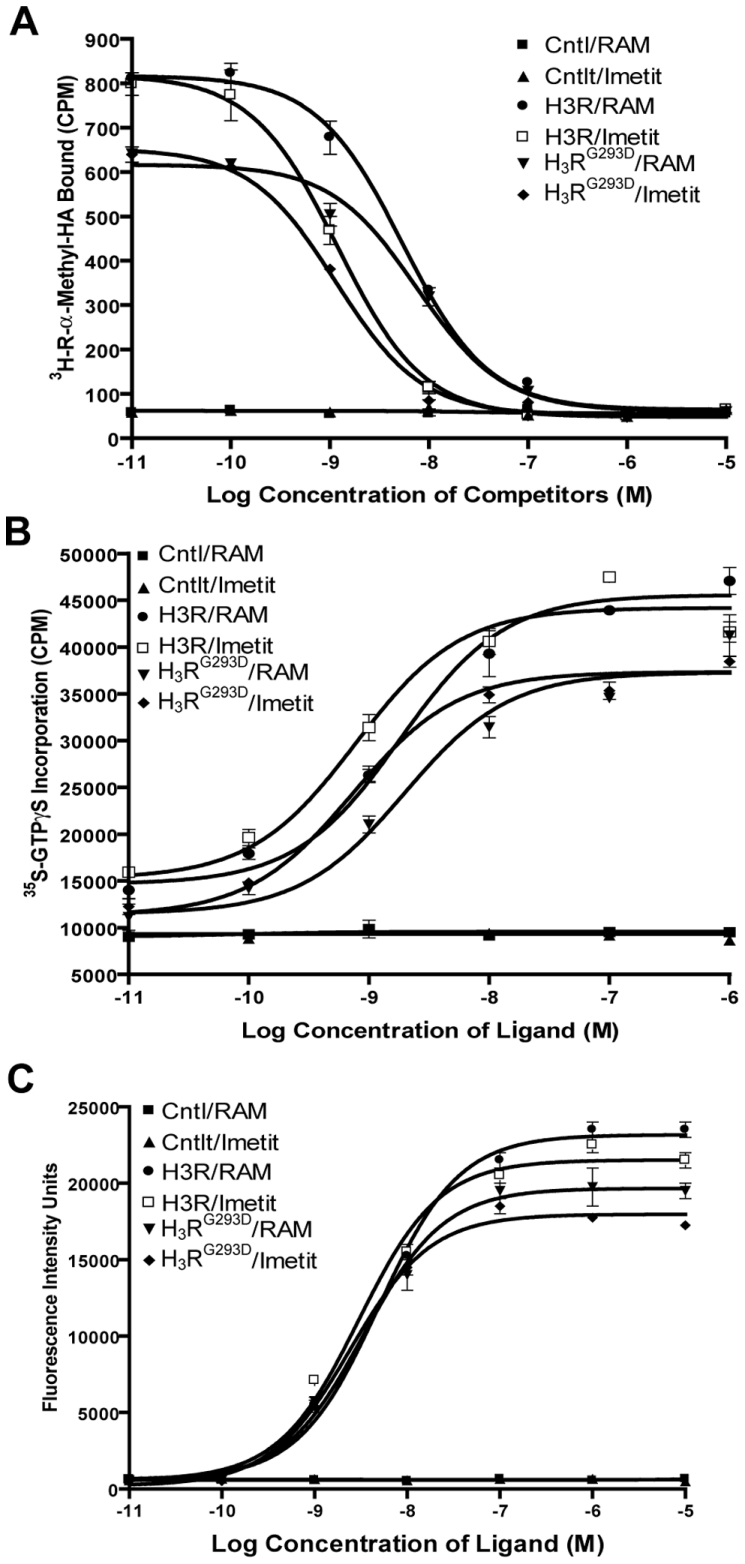
Pharmacological comparison of *Hrh3* allele functionality. (**A**) Characterization of H_3_R and H_3_R^G293D^ using radioligand binding assays. Plasmids carrying *Hrh3* cDNA for the indicated alleles were transiently transfected into COS-7 cells. Cell membranes from transfected cells were used in radioligand binding assays with 3H-RAM-HA as the tracer in the presence of various concentrations of unlabeled RAM-HA and Imetit as the competitors (see Materials and Methods). Membranes from mock transfected COS-7 cells were used as the negative controls (Cntl). (**B**) Functional comparison of H_3_R and H_3_R^G293D^ in GTPγS binding assays. Membranes from CHO cells transiently expressing H_3_R cDNA for the indicated alleles were stimulated with various concentrations of RAM-HA and Imetit in the presence of 35S-GTPγS as the tracer (see Materials and Methods). Membranes from mock transfected CHO cells were used as the negative control (Cntl). (**C**) Functional comparison of H_3_R and H_3_R^G293D^ Ca2+ mobilization assays. 293T cells co-expressing Gqi5 and H_3_R or H_3_R^293D^ were stimulated with different concentrations of RAM-HA and Imetit. The ligand-stimulated Ca^2+^ mobilization was monitored using a FLIPR instrument (Molecular Device) (see Materials and Methods). 293T cells expressing Gqi5 alone were used as controls (Cntl).

To assess the functionality of the G293D polymorphism, we expressed the two alleles and compared the pharmacologic properties of the H_3_R ligands R-α-methylhistamine (RAM-HA) and Imetit in a radioligand binding assay, a GTPγS-binding assay, and a ligand-induced Ca^2+^ mobilization assay (**Fig. 2A–C**). Overall, no significant difference in either receptor affinity or activity was detected, suggesting that the G293D polymorphism does not alter the function of the protein per se. However, these results do not exclude the possibility that an *Hrh3* isoform expression polymorphism may underlie *Eae8,* since the assays described above are limited to utilizing full length *Hrh3* cDNA expressed under a heterologous promoter.

### Differential Hrh3 Isoform Expression in SJL and B10.S Mice in Response to Peripheral Inflammatory Stimuli

Multiple H_3_R isoforms have been described for humans, rats, and mice. In the human, H_3_R isoforms demonstrate differences in pharmacologic activity [33], [34], [35]. Many of these isoforms differ from the full length transcript by a variable-length deletion in the third IC loop. Importantly, isoform variation in this region results in differences in H_3_R functional activity. For example, an 80 amino acid deletion at the third IC loop of human H_3_R confers increased constitutive activity [21]. In the rat, 32 and 48 amino acid deletions, which in the mouse encompasses the G293D polymorphism, result in changing H_3_R’s efficiency in G protein coupling to second messenger signaling pathways [19], [20]. Consequently, these deletions result in increased constitutive activity, similar to the 80 amino acid deletion in the human H_3_R.

In our previous EAE study, H_3_RKO mice immunized for the induction of EAE exhibited increased BBB permeability on D4 post-immunization, significantly earlier than the appearance of inflammatory cells in the CNS [11]. This finding supports a role for events predicted by the neural-initiated disease hypothesis, which posits that events within the CNS initiate the disease process and influence subsequent autoimmune responses. We reasoned therefore that differential expression of H_3_R isoforms in response to inflammatory stimuli i.e., adjuvants/pain/danger signals etc., may be important in neurogenic control of disease susceptibility in SJL and B10.S mice. In this regard, both pertussis toxin (PTX) [36] and complete Freund’s adjuvant (CFA) [37] lead to increased BBB permeability and systemic exposure to lipopolysaccharide (LPS) directly disrupts endothelial cell barrier functions [38] including BBB transport of amyloid proteins [39], [40], which have recently been shown to suppress EAE severity [41]. Moreover, advances in neuroscience and immunology have established the anatomical and cellular basis for bidirectional communication between the nervous system and immune systems [22]. To explore the possibility that inflammatory stimuli can impact *Hrh3* isoform expression, *Hrh3* isoform expression was studied by RT-PCR using forebrain tissue from untreated SJL and B10.S mice, or at D1 and D10 after immunization with proteolipid protein peptide 139–151 (PLP_139–151_)+ complete Freund’s adjuvant (CFA)+pertussis toxin (PTX), or with each of the respective components of the adjuvants used to induce disease, CFA, PTX, or CFA+PTX. Isoform-specific RT-PCR primers were designed based on the 3 published rat isoform sequences; thus detecting murine orthologs of the rat *Hrh3a* (full length transcript), *Hrh3b* (missing 32 amino acids from the third IC loop), and *Hrh3c* (missing 48 amino acids from the third IC loop) isoforms [19], [20].

**Figure 3:**
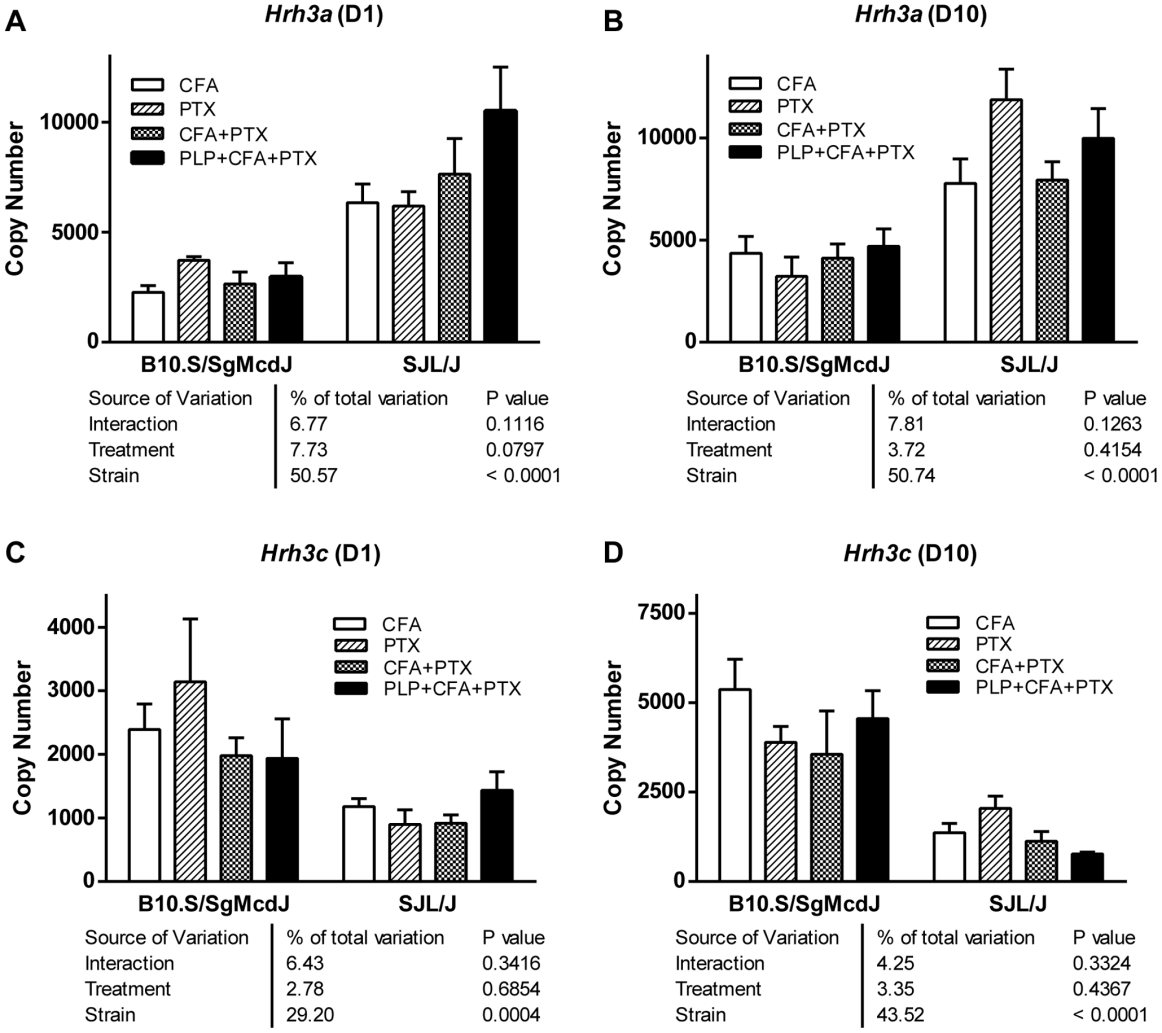
*Hrh3* isoform expression is influenced primarily by strain. Expression levels were determined by real time PCR using mRNA isolated from perfused forebrains on D1 (A) and (C) and D10 (B) and (D) post-injection with either CFA, PTX, CFA+PTX or PLP_139–151_+CFA+PTX, as described in Materials in Methods (n = 4–8). Data were analyzed by two-way ANOVA for effect of treatment and strain.

**Figure 4:**
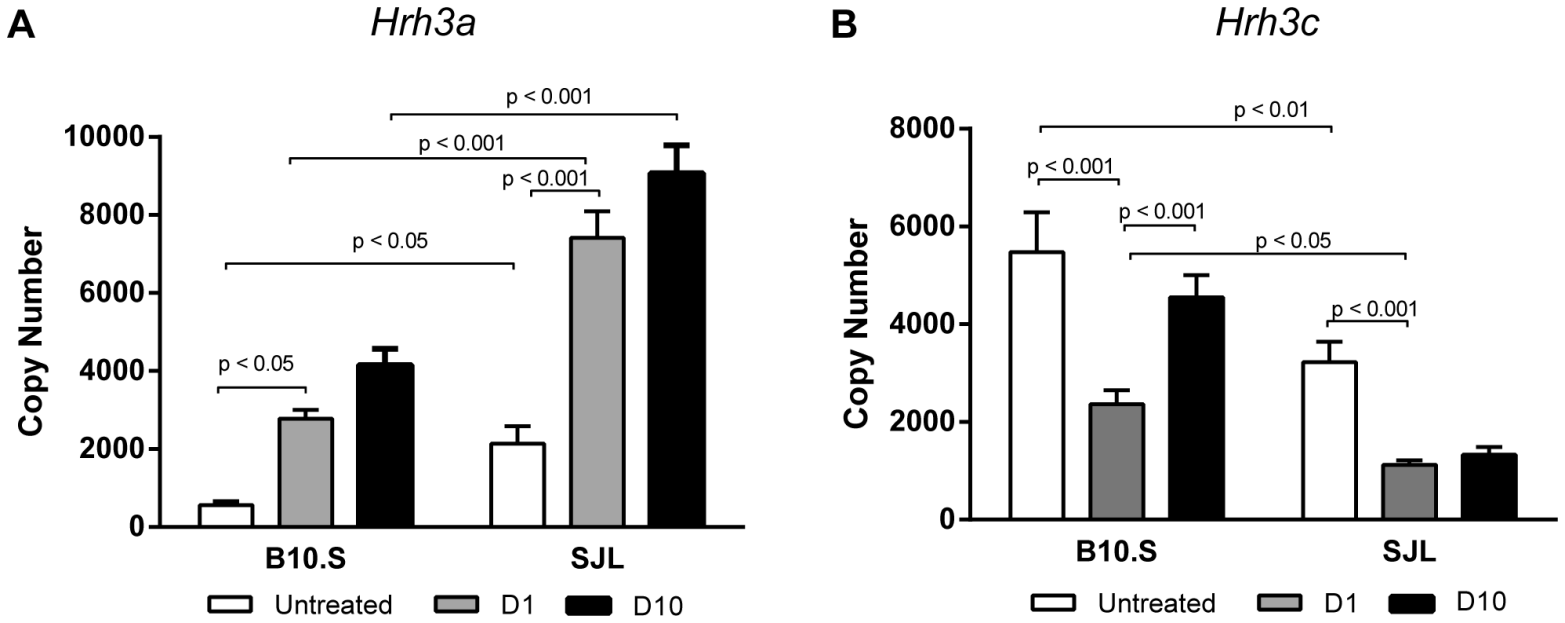
Differential *Hrh3* isoform expression in B10.S and SJL mice in response to peripheral inflammatory stimuli. (**A**) *Hrh3a* expression in the forebrain of B10.S and SJL mice (n = 20), as determined by qRT-PCR (see Materials and Methods), either untreated, or D1 and D10 post-treatment. (**B**) *Hrh3c* expression in the forebrain of B10.S and SJL mice, untreated, or D1 and D10 post-treatment. Data were analyzed by two-way ANOVA for effect of treatment and strain.

*Hrh3b* in whole forebrain was consistently below the level of detection in both B10.S and SJL mice irrespective of treatment; in contrast, *Hrh3a* and *Hrh3c* transcripts were readily quantifiable. For both detectable isoforms, there was a response to treatment, but no difference between the four inflammatory stimuli, and no strain-by-treatment interaction, indicating that strain is the major source of variation underlying differential expression of the two isoforms (**Fig. 3**). Consequently, the treatment groups were pooled by strain and reanalyzed (**Fig. 4**).

Basal expression of *Hrh3a* was higher in SJL forebrain compared to B10.S, whereas *Hrh3c* was lower (**Fig. 4**). Treatment resulted in an increase in *Hrh3a* expression, and a decrease in *Hrh3c* expression in both strains. Expression of *Hrh3a* remained elevated between D1 and D10 in both strains; however, it remained higher in SJL mice compared to B10.S (**Fig. 4A**). In contrast, *Hrh3c* expression increased from D1 to D10 in B10.S mice, whereas it remained low in SJL mice (**Fig. 4B**). Overall, these results support the existence of both a basal and a treatment-specific *Hrh3* isoform expression polymorphism.

Our previous findings with H_3_RKO mice suggested that signaling through H_3_R may be protective in EAE. Although EAE-susceptible SJL mice tend to express higher levels of the full length *Hrh3a* isoform, they express lower levels of the short *Hrh3c* isoform relative to B10.S mice. Given the finding that the rat ortholog of *Hrh3c* is constitutively active [19], [20], and assuming that the mouse and rat orthologs are functionally homologous, we suggest that constitutive signaling through *Hrh3c* is protective in EAE. Consistent with this, expression of the *Hrh3c* isoform was not upregulated in SJL mice by D10, whereas it was upregulated in B10.S mice (**Fig. 4B**). Taken together, our findings suggest that differential *Hrh3* isoform expression in response to peripheral inflammatory stimuli regulates neurogenic control of EAE in SJL and B10.S mice, and is a potential functional candidate polymorphism underlying *Eae8*. We also acknowledge the possibility that other candidate genes in addition to *Hrh3* may reside within the *Eae8* locus, particularly given the somewhat modest difference in *Hrh3* isoform expression between SJL and B10.S mice (Fig. 4). However, since the *Hrh3* alternative splicing and expression is regulated very rapidly after inflammatory insult (Fig. 4), we believe that modest changes in expression early can significantly alter the course of the subsequent immune response, and can lead more profound modulation of disease course later. For example, 2 fold overexpression of Tlr7 due to a gene translocation event is sufficient to significantly accelerate the course of autoimmune lupus [42], [43].

The candidacy of *Hrh3* for *Eae8* is further supported by comparing the functional roles of H_3_R and their relationship to the clinical presentations of MS and EAE. H_3_R is expressed presynaptically where it is an inhibitory autoreceptor and heteroreceptor [44]. Consequently, the neurophysiologic roles of H_3_R are complex and impact a variety of phenotypes including weight, metabolism, cognition/memory, arousal, sensory-motor activity, thermoregulation, and inflammatory and non-inflammatory pain [44]. Many, if not all of these are dysregulated in MS [45], [46], [47], [48]. For example, ∼50% of MS patients experience one or more types of pain simultaneously, occurring at any point during the disease course [45]. In SJL/J mice with either EAE or Theiler’s murine encephalomyelitis virus (TMEV) induced demyelination, a viral model of MS, dysregulated pain sensation, including allodynia and hyperalgesia, is observed [49], [50], [51]. In EAE, these effects involve dysregulation of the glutamatergic system [50] in which H_3_R, as a presynaptic heteroreceptor, negatively modulates glutamate release [44]. Similarly, cognitive impairment which is common in MS [46], [52] is also seen in EAE in association with dysregulated glutamatergic and GABAergic transmission [53].

### Hrh3 Expression in Secondary Lymphoid Tissues and by Hematopoietic Cells

In our earlier EAE study [11], and in a study on cerebral malaria in mice [14], it was proposed that H_3_R plays a role in neurogenic control of T cell effector responses. As such, *Hrh3* would serve as a key gene in the elaborate interactions between the brain and the immune systems [54] comprising a neural inflammatory reflex [22], [23], [24]. To exclude the possibility that H_3_R is influencing immune responses directly through its expression in either secondary lymphoid tissues and/or hematopoietic cells including T cells, we examined its expression in the spleen and LN of naive animals, and by macrophages, mast cells, neutrophils, bone marrow-derived dendritic cells, B cells (B220+), effector CD8+ (TCRβ+CD8+CD4−), naive CD4+ (TCRβ+CD4+CD8−CD25^low^CD45R^high^CD44^low^CD1d-tetramer-), memory CD4+ (TCRβ+CD4+CD25^low^CD45RB^low^CD44^high^CD1d-tetramer-), NKT (CD1d tetramer+), and Treg cells (TCRβ+CD4+Foxp3+) by RT-PCR. mRNA for any of the known *Hrh3* isoforms was undetectable in both whole spleen and LN, as well as in all cells of the innate and adaptive immune systems studied. These data are also predicted by the neural-initiated disease hypothesis and identify H_3_R as a key CNS intermediate in a neural inflammatory reflex influencing T cell effector responses and susceptibility to EAE, presumably through innervation of secondary lymphoid tissues.

**Figure 5:**
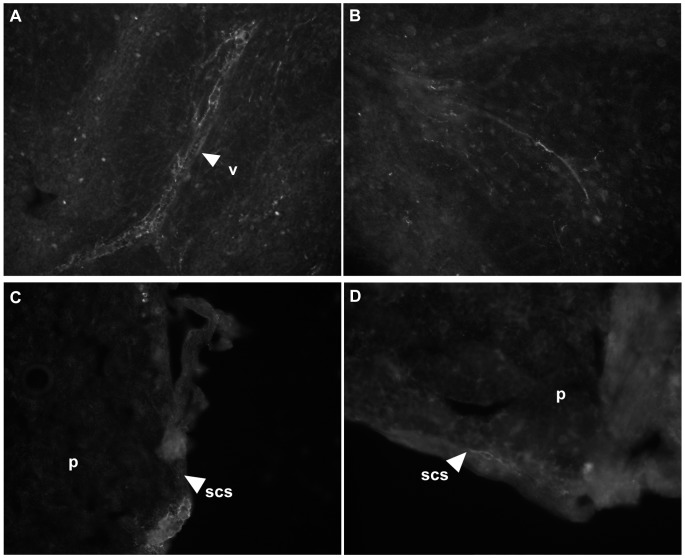
Spleen and LN are innervated at physically relevant locations. Fixed, frozen sections of spleen (A and B) and pancreatic LN (C and D) from C57BL/6 mice were stained with anti-NSE antibody and visualized with Cy3-conjugated secondary antibody. (A) and (B), 200×magnification; (C) and (D), 400×magnification. Figure labels: v, vascular plexus; scs, subcapsular sinus; p, parenchyma.

To confirm the innervation of mouse spleen and LN, we employed immunohistochemistry using neuronal-specific enolase (NSE), a pan-neuronal marker [55]. In the spleen, dense innervation was observed, particularly around the vasculature, as previously reported [56] (**Fig. 5A and B**). However, in contrast to what has been reported for the LNs of other species, where nerve fibers have been shown to branch into the parenchyma in paracortical and cortical regions [57], only sparse innervation was detected in the mouse LN. This was primarily located in the capsular and subcapsular sinus (SCS) (**Fig. 5C and D**) and absent from the parenchyma. This pattern of innervation was similar regardless of the age of the mouse or LN location (data not shown). Although in our hands the mouse LN lacked innervation in traditional T and B cell zones, the finding of innervation in the capsular and SCS is nevertheless consistent with the finding that particulate antigens and pathogens arriving via the lymphatics are retained in the SCS of mouse LN **[58]**, [59], [60], [61], **[62]**, and that naive T cells can relocalize to the SCS in response to infection [63]. Moreover, SCS macrophages can present antigen to B cells [59], [61], [62], in line with their ability to retain antigen on their surface, rather than internalize and degrade it [64], [65]. Additionally, SCS macrophages have been shown to be specialized APCs, which in conjunction with non-cognate B cells deliver opsonized antigens to germinal centers thereby promoting affinity maturation [66].

Taken together, our results support a role for innervation of secondary lymphoid tissues in H_3_R-mediated neurogenic control of immune responses. Nevertheless, it is worth noting that H_3_R-mediated neurogenic control of immune responses via the hypothalamic-pituitary-adrenal axis cannot be completely excluded in EAE since H_3_R agonist stimulated release of adrenocorticotropic hormone (ACTH) from the mouse pituitary tumor cell line, AtT-20, can be blocked by first generation H_3_R antagonists [67], [68]. However, a recent study brings into question the role of H_3_R in regulating ACTH release. Using a highly selective second generation antagonist capable of pharmacologically discriminating H_3_R and H_4_R indicates that H_4_R rather than H_3_R regulates ACTH release from AtT-20 cells [69].

### H_3_R Signaling Regulates Gene Expression in LN

Given the findings that H_3_R negatively regulates the development of EAE, a T cell-mediated autoimmune disease, that antigen-specific CD4^+^ T cells from H_3_RKO animals demonstrate a unique effector profile [11], [14], and that the microenvironments of secondary lymphoid tissues are anatomically poised to be subjected to H_3_R mediated neurogenic control, we evaluated the possibility that central H_3_R signaling modulates gene expression in the LN under basal conditions and in early responses to peripheral stimuli. To this end, we utilized microarrays to examine baseline gene expression in the LN of untreated WT and H_3_RKO mice. Because changes in *Hrh3* isoform expression occur within 24 hours of exposure to peripheral inflammatory stimuli (**Fig. 4**), we also examined gene expression at 24 hours following the administration of the two adjuvants used to induce EAE, CFA alone or in combination with PTX.

Relatively few differences in gene expression between WT and H_3_RKO mice were observed in the LN at baseline (9 genes, **Table 1**). However, most of these genes were of immunological relevance, as identified by Ingenuity® Pathway Analysis (IPA). Several immunoglobulin (Ig) genes were downregulated in H_3_RKO LN, whereas S100 calcium-binding proteins A8 and A9 (*S100a8* and *S100a9*) were upregulated. The latter play an important role in inflammatory processes, and are upregulated during MS and EAE [70].

**Table 1:** Genes differentially expressed in WT vs. H_3_RKO LN at baseline.

p-value	FC	Symbol	Entrez Gene Name
1.03E-03	−10.1	Mup1	major urinary protein 1
8.11E-03	−2.70	IGHG1	immunoglobulin heavy constant gamma 1 (G1m marker)
3.99E-03	−2.57	IGHM	immunoglobulin heavy constant mu
7.18E-03	−2.37	RETN	resistin
3.16E-03	−2.14	CFD	complement factor D (adipsin)
2.46E-03	2.07	FPR2	formyl peptide receptor 2
2.36E-02	2.10	VCAN	versican
1.17E-02	2.32	CLEC4D	C-type lectin domain family 4, member D
1.18E-02	2.90	S100A8	S100 calcium binding protein A8
4.40E-02	3.14	Chi3l3/Chi3l4	chitinase 3-like 3
9.02E-03	3.30	S100A9	S100 calcium binding protein A9

Gene expression was determined by microarray analysis. The criterion for differential expression was set at p<0.05 and signed fold change >2. Fold change (FC) indicates the change in expression in H_3_RKO LN relative to WT.

CFA treatment resulted in 13 differentially expressed genes between WT and H_3_RKO LN (**Table 2**), many of which were also associated with inflammatory responses. Similar to baseline, several Ig genes remained lower in H_3_RKO LN after CFA treatment.

**Table 2:** Genes differentially expressed in WT vs. H_3_RKO LN after CFA treatment.

p-value	FC	Symbol	Entrez Gene Name
2.09E-03	−3.39	IGHA1	immunoglobulin heavy constant alpha 1
1.21E-02	−3.14	IGHG1	immunoglobulin heavy constant gamma 1 (G1m marker)
7.87E-03	−2.58	IGHM	immunoglobulin heavy constant mu
1.26E-02	−2.52	Mup1	major urinary protein 1
4.42E-02	−2.43	GDA	guanine deaminase
4.65E-02	2.04	RBP4	retinol binding protein 4, plasma
4.68E-02	2.12	PCOLCE2	procollagen C-endopeptidase enhancer 2
4.82E-02	2.34	NPR3	natriuretic peptide receptor C/guanylate cyclase C
2.54E-02	2.61	Aldh1a7	aldehyde dehydrogenase family 1, subfamily A7
1.15E-02	2.82	PPBP	pro-platelet basic protein (chemokine (C-X-C motif) ligand 7)
3.39E-03	3.05	TMEM45B	transmembrane protein 45B
2.48E-02	3.23	NNAT	neuronatin
3.11E-02	5.92	Xlr3c	X-linked lymphocyte-regulated 3C

Gene expression was determined by microarray analysis. The criterion for differential expression was set at p<0.05 and signed fold change >2. Fold change indicates the change in expression in H_3_RKO LN relative to WT.

**Figure 6:**
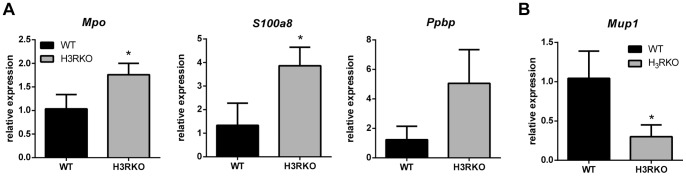
qRT-PCR validation of genes differentially expressed in WT and H_3_RKO LN upon CFA+PTX treatment. WT and H_3_RKO (n = 3 for WT, and n = 4 for H_3_RKO) mice were injected with CFA+PTX. RNA was isolated from LN 24 hours later and subjected to qRT-PCR analysis (see materials and methods) to quantify the mRNA expression of the indicated genes. Data were analyzed using unpaired parametric T test. * indicates p<0.05 compared to WT.

The greatest differences in gene expression between WT and H_3_RKO LN were observed after treatment with CFA+PTX, the EAE immunization protocol that elicits increased disease severity in H_3_RKO mice compared to WT. There were 29 genes differentially expressed between the strains (**Table 3**). Consistent with increased EAE severity, the results of IPA revealed that many of the differentially expressed genes were involved in inflammation, with a Z-score clearly indicative of an exaggerated inflammatory/immune response in the LNs of H_3_RKO animals following exposure to CFA+PTX (**Tables 4** **and** **5**). Most genes associated with inflammatory functions were overexpressed in H_3_RKO animals, including Ig genes, which were expressed at lower levels at baseline, and *S100a8* and *S100a9*, which were expressed at higher levels at baseline. We selected three pro-inflammatory genes, *S100a8*, pro-platelet basic protein/chemokine (C-X-C motif) ligand 7 (*Ppbp*), and myeloperoxidase (*Mpo*), all of which were expressed at higher levels in H_3_RKO LN after CFA+PTX treatment (*Ppbp* was also overexpressed in H_3_RKO LN after CFA treatment), for validation by qRT-PCR in a series of independent experiments. We confirmed that *S100a8* and *Mpo* were expressed at significantly higher levels in H_3_RKO LN, while expression of *Ppbp* showed an increase that did not reach statistical significance (**Fig. 6A**). Furthermore, we also confirmed downregulation of *Mup1*, a gene that was under-expressed in H_3_RKO LN (**Fig. 6B**). Interestingly, *S100a8*, *Mpo*, and *Ppbp* are differentially expressed in the liver of CBA and BALB/c mice in response to Schistosoma infection, with higher expression correlating with more severe pathological outcome [71]. Taken together, given the exaggerated expression of inflammation-related genes in H_3_RKO LN, these results are consistent with negative regulation of peripheral immune responses by central H_3_R signaling.

**Table 3:** Genes differentially expressed in WT vs. H_3_RKO LN after CFA+PTX treatment.

p-value	FC	Symbol	Entrez Gene Name
1.75E-03	−12.3	Mup1	major urinary protein 1
3.24E-03	−2.90	CFD	complement factor D (adipsin)
4.97E-02	−2.83	FOXA1	forkhead box A1
1.03E-02	−2.76	RETN	resistin
1.24E-02	−2.36	Serpina3k	serine (or cysteine) peptidase inhibitor, clade A, member 3K
8.85E-03	−2.27	H19	H19, imprinted maternally expressed transcript
6.27E-03	−2.27	STK31	serine/threonine kinase 31
1.42E-03	−2.14	ITIH4	inter-alpha (globulin) inhibitor H4
3.17E-02	−2.13	CITED1	Cbp/p300-interacting transactivator
5.21E-03	−2.06	ADRB3	adrenergic, beta-3-, receptor
3.12E-02	2.02	IGHG1	immunoglobulin heavy constant gamma 1
3.42E-02	2.04	FOS	FBJ murine osteosarcoma viral oncogene homolog
1.43E-02	2.25	ACOT2	acyl-CoA thioesterase 2
4.61E-02	2.32	IGHM	immunoglobulin heavy constant mu
1.76E-02	2.39	SLPI	secretory leukocyte peptidase inhibitor
3.53E-02	2.39	PRTN3	proteinase 3
1.85E-02	2.42	ELANE	elastase, neutrophil expressed
4.37E-02	2.60	IGHA1	immunoglobulin heavy constant alpha 1
2.41E-02	2.62	Beta-s	hemoglobin subunit beta-1-like
2.04E-03	2.75	CTSG	cathepsin G
4.33E-02	2.79	Ngp	neutrophilic granule protein
2.46E-02	2.80	S100A8	S100 calcium binding protein A8
4.39E-02	3.14	Igk-v19-14	immunoglobulin kappa variable 6-14
1.15E-02	3.31	Acot1	acyl-CoA thioesterase 1
1.64E-02	3.48	S100A9	S100 calcium binding protein A9
1.57E-02	3.80	MPO	myeloperoxidase
4.90E-03	4.96	PPBP	pro-platelet basic protein (chemokine (C-X-C motif) ligand 7)

Gene expression was determined by microarray analysis. The criterion for differential expression was set at p<0.05 and signed fold change >2. Fold change indicates the change in expression in H_3_RKO LN relative to WT.

**Table 4:** Top biological pathways associated with changes in inflammation-associated gene expression in H_3_RKO vs. WT LN after CFA+PTX treatment.

Category	p-value	Functions Annotation	PAS^a^	z-score	Molecules
*Inflamm. Response*	*2.57E-07*	*inflammatory response*	*Increased*	*2.24*	*9*
Inflamm. Response	9.19E-08	cell movement of neutrophils	Increased	2.13	7
Inflamm. Response	1.11E-06	chemotaxis of neutrophils		1.84	5
Inflamm. Response	8.32E-08	immune response		1.76	13
Inflamm. Response	1.63E-06	adhesion of neutrophils		1.69	4
Inflamm. Response	4.53E-07	cell movement of phagocytes		1.63	8
Inflamm. Response	2.31E-07	Inflammation		0.30	8
Inflamm. Response	1.91E-07	activation of leukocytes		0.15	9

Results of IPA run on genes differentially expressed between WT and H_3_RKO LN after CFA+PTX treatment. The top 8 associated biological pathways are shown. ^a^PAS, predicted activation state.

**Table 5:** Predicted effects on inflammatory response function in H_3_RKO vs. WT LN after CFA+PTX treatment.

Genes in dataset	Prediction	Fold Change	Findings
PPBP	Increased	4.96	Increases (15)
ELANE	Increased	2.42	Increases (1)
S100A8	Increased	2.80	Increases (8)
S100A9	Increased	3.48	Increases (9)
PRTN3	Increased	2.39	Increases (2)
CTSG	Increased	2.75	Increases (10)
MPO	Increased	3.80	Increases (1)
SLPI		2.39	Affects (2)
FOS		2.04	Affects (1)

Molecules differentially expressed in the pathway with the highest Z-score in Table 4, showing gene expression changes for individual molecules and the predicted impact on the associated annotated function (inflammatory response).

In summary, our studies suggest that an *Hrh3* isoform expression polymorphism regulates neurogenic control of EAE and T cell effector responses in mice, and is a potential functional candidate polymorphism underlying *Eae8*. Moreover, our data, predicted by the neural-initiated disease hypothesis, identify H_3_R as a key intermediate in a neural immune reflex [22], [23], [24] integrating peripheral inflammatory signals in the neurogenic control of disease susceptibility and T cell effector responses. Importantly, our findings which functionally unite the opposing neural-initiated and immune-initiated theories underlying neuroinflammatory reactions in MS provide potential insight into the mechanisms whereby gene-by-environmental stimuli may determine the long term progression and spectrum of clinical disease courses seen in MS [25] associated with subtle changes in BBB integrity preceding inflammatory lesions [72], [73]. Pharmacologic targeting of H_3_R may therefore be useful in preventing the development and formation of new lesions in MS, thereby significantly limiting the progress of the disease.

## Materials and Methods

### Animals

C57BL/6J, B10.S/SgMcdJ and SJL/J mice were purchased from the Jackson Laboratory (Bar Harbor, ME). B6.129P2-*Hrh3^tm1Twl^* mice [74] (H3RKO), originally held at Johnson and Johnson Pharmaceutical Research and Development (San Diego, CA) were maintained at the University of Vermont (Burlington, VT). B10.S.*eae8^SJL^* mice were generated by marker assisted selection using informative microsatellite markers spanning the *eae8* interval [11]. Animals were backcrossed for ten generations at which point they were intercrossed and subsequently fixed as a homozygous interval-specific congenic line. All animals were maintained under specific pathogen free conditions on a 12∶12 light dark cycle and were fed Purina mouse pellets (Ralston-Purina, St. Louis, MO) and water ad libitum. The experimental procedures performed in this study were approved by the Institutional Animal Care and Use Committee (IACUC) of the University of Vermont; IACUC protocol number 08–034, approved November 9, 2007. Guinea pig samples were obtained in a previously published study [75].

### Identification of Polymorphisms in Hrh3

Total RNA from SJL/J and B10.S/SgMcdJ was isolated from adult spleen. cDNAs were PCR amplified using Taq polymerase and specific primer pairs flanking the mRNA coding region of *Hrh3*. The amplified fragments were cloned and at least three clones for each PCR fragment were sequenced from both insert termini. A single nucleotide polymorphism at position 878 (G→A) leading to a single amino acid change from glycine to asparagine at residue 293 (G293D) in the predicted sequence of H3R was identified between B10.S/SgMcdJ and SJL/J allele. Multiple sequence alignment was done using the MultAlign website [63]. Rat *Hrh3* sequence was obtained from NCBI, accession number NP_445958.

To detect the 878 (G→A) *Hrh3* SNP in different inbred strains RFLP was used. oligonucleotide primers flanking the 878 (G→A) SNP were designed: forward primer: 5′-CAAGACGGGCTGTTCGG-3′; reverse primer: 5′-TCACGATGATAGCCA GCGA CTT-3′. The presence of the 878 (G→A) SNP gives rise to a FokI restriction site that distinguishes the two alleles: B10.S/SgMcdJ→661 bp with codon GGT→27, 36, 68, 126, 403 bp fragments; SJL/J→661 bp with codon GAT→27, 36, 68, 126, 177, 226 bp fragments.

### Functional Characterization of Hrh3 Alleles

Radioligand competition binding assays were performed essentially as described [76]. Briefly, *Hrh1* and *Hrh1* ^G293D^ expression plasmids were transfected into COS-7 cells using Lipofectamine (Invitrogen, Carlsbad, CA). Membranes were prepared in binding buffer (50 mM Tris-HCl, pH 7.5, 5 mM EDTA). ^3^H-R-α-methyl-histamine (RAM-HA) was used as the tracer at a final concentration of 1 nM. Unlabeled RAM-HA and Imetit at various concentrations were added as the competitors. The binding assays were carried out at room temperature for 1 hour. The binding mix was then filtered through 96-well GFC (Packard Instrument, Meriden, CT) filter plates and the plates were washed with ice cold binding buffer and dried in a 50^o^C oven. After adding 50 µl Microscint-0 (Packard Instrument, Meriden, CT) to each well of the 96-well filters, the filters were counted in TopCount/NTX (Packard Instrument, Meriden, CT) to measure the bound ^3^H-RAM-HA.

GTPγS binding assays were essentially performed as described [77]. The mouse *Hrh3* and *Hrh3* ^G293D^ expression plasmids were transiently transfected into CHO cells using Lipofectamine. Two days after transfection, the membranes were prepared from the transfected cells and GTPγS binding was performed using either RAM or Imetit as the stimulator.

Ca^2+^ mobilization assays were carried out using the *Hrh3* and *Hrh3* ^G293D^ variants co-transfected into 293T cells with Gq_i5_, a chimeric G-protein that shifts cAMP inhibition to Ca^2+^ mobilization, respectively. 293T cells transfected with Gq_i5_ alone were used as the control. Two days after transfection, the transfected cells were seeded into 96-well black poly-D-lysine coated tissue culture plates and loaded with Fluo-3. Ligand-stimulated Ca^2+^ mobilization was monitored using FLIPR (Molecular Devices, Sunnyvale, CA).

### Quantification of Hrh3 Isoform Expression

Mice were immunized with either (1) CFA; (2) CFA and 200 ng of PTX administered by i.v. injection immediately after the CFA injection; (3) 200 ng of PTX administered by i.v. injection or (4) 200 µg of PLP_139–151_ emulsified in CFA and 200 ng of PTX by i.v. injection immediately thereafter. All CFA injections were administered by equally distributing 0.1 ml emulsion over three sites (left and right flank and base of the tail). Mice were sacrificed on D1 and D10 post immunization.

For tissue collection, mice were anesthetized using Ketaset (Fort Dodge, IA) and perfused with 20 ml of phosphate buffer saline (PBS). Brain samples were snap frozen in liquid nitrogen and stored at −80°C until further processed for RNA isolation. Total RNA was extracted using RNeasy kit followed by a DNase treatment (Qiagen, Valencia, CA) according to the manufacturer’s guidelines. The reverse transcription of RNA was performed using the Superscript III RT kit (Invitrogen, Carlsbad, CA).

Probes for the three *Hrh3* isoforms were designed using the Primer Extension software (Applied Biosystems, Foster City, CA). All real-time PCR reactions were performed using an ABI prism 7900 HT sequence Detection system with the sequence detection software system SDS 2.2 in accordance with the manufacturer’s instructions, using Taqman chemistry. Standard curve assay, using serially diluted *Hrh3* isoform-specific plasmid clones as standards, was used to determine copy number of *Hrh3* isoforms in the samples. The copy number was then normalized to the internal control gene m*HPRT*. qPCR amplification efficiencies for *Hrh3a, Hrh3b, Hrh3c* isoform-specific primer sets were 92.5, 96.2, and 108.1%, respectively, with r^2^ values = 0.99.

### Hrh3 Expression by Cells of the Innate and Adaptive Immune Systems

Alveolar macrophages were collected by lavaging the lungs through a tracheal cannula with 1 ml DPBS from which cells were collected, counted by hemocytometer, and differential analysis was performed by cytospin and H&E stain. Nearly 100% of the cells were identified as alveolar macrophages in these preparations.

For the generation of bone marrow-derived dendritic cells (BMDC), bone marrow was flushed from the femurs and tibiae and cultured on 24-well plates at 1×10^6^ cells/well (1 ml/well) in RPMI-1640 containing 10% serum and 10% conditioned media from X63-GMCSF myeloma cells transfected with murine GM-CSF cDNA (kindly provided by Dr. Brent Berwin, Dartmouth College). Media was replaced on days 2 and 4 and the adherent and lightly-adherent BMDCs, predominantly CD11b^+^CD11c^+^ by FACS, were collected on day 6.

For the preparation of neutrophils, the marrow was flushed from femurs and tibias with HBSS, layered atop a three-step Percoll gradient (72, 64, and 52%), and centrifuged at 1,060×*g* for 30 minutes. Samples of the 72∶64% interface revealed greater than 95% morphologically mature-appearing neutrophils.

Resident mast cells were collected by lavaging the peritoneal cavity five times using 5ml of DPBS per lavage. The cells were layered atop a 75% Percoll solution and centrifuged at 600×*g* for 15 minutes at room temperature. Mast cells in the pellet were visualized by cytospin to be greater than 90% pure. Naïve CD4^+^ (TCRβ^+^CD4^+^CD25^−^CD45RB^High^CD44^Low^CD1d-tetramer^–^), memory CD4^+^ (TCRβ^+^CD4^+^CD25^−^CD45RB^Low^CD44^High^CD1d-tetramer^–^), and NKT (CD1d-tetramer^+^) cells were sorted using the following surface anti-mouse mAb: (H57-5987, eBioscience); anti-CD4 (MCD0417, Caltag); anti-CD25, anti-CD44, and anti-CD45RB (PC61, IM7, 16A; BD Pharmingen). CD1d tetramer was kindly provided by NIH. Sorted cells were kept at −80°C for subsequent RNA extraction.

Total RNA was extracted using an RNeasy isolation kit (Qiagen Inc.). mRNA was reverse transcribed using Superscript III reverse transcriptase (Invitrogen). The generated cDNA was used in qRT-PCR using an *Hrh3* probe (Mm 00446706_m1; Applied Biosystems). β2-microglobulin and *Hprt* were used as reference genes and the relative expression levels were calculated using the comparative threshold cycle (C_T_) method.

### Microarray Analysis

Microarray analysis was conducted on female and male C57BL/6 WT or H_3_RKO mice at 8 weeks of age. Mice in the treatment groups were injected with either with CFA or CFA+PTX and euthanized at 24h. LN were removed from C57Bl/6 WT and H_3_RKO mice at 8 weeks, snap frozen in liquid nitrogen. Isolation and purification of RNA was completed using the RNeasy RNA extraction kit (Qiagen).

RNA amplification and microarray analysis was performed at UVM microarray core facility using manufacturer’s described protocols [78]. Briefly, 2 ug of total RNA from each sample were reverse transcribed to the single stranded cDNA using T7-oligo(dT) primer. T4 DNA polymerase was used to synthesize double-stranded cDNA, which served as a template for *in vitro* transcription using T7 RNA polymerase to produce biotinylated cRNA. The biotinylated cRNAs were fragmented into 50- to 200-base fragments and then hybridized to GeneChip Mouse Genome 430A 2.0 Arrays for 16 h at 45°C in a rotating Affymetrix GeneChip Hybridization Oven 320. After hybridization, arrays were washed and stained with streptavidin-phycoerythrin on an automated Affymetrix GeneChip Fluidic Station F450. The arrays were scanned with an Affymetrix GeneChip Scanner 2700 and the images quantified using Affymetrix GeneChip Operating Software.

The signal intensity for each probe on each chip was calculated from scanned images using GeneChip Operating Software (Affymetrix), and signal intensities were analyzed using BioConductor (http://www.bioconductor.org). Probe intensities were background corrected, normalized, and summarized using the Robust Multichip Average algorithm described by Speed and coworkers [79], [80], including background-correction, normalization, and summarization for each probe set and sample, using Partek Genomic Suites® version 6.6 (Copyright © 2009, Partek Inc., St. Louis, MO, USA). Microarray datasets were uploaded to the Gene Expression Omnibus repository, accession number GSE44873.

Sample quality was assessed based on the 3′:5′ ratio, relative log expression, and normalized unscaled standard error. Principal Component Analysis was used to screen for outlier samples that could potentially introduce latent variation into the analysis of differential expression across sample groups (none were detected).

To identify differentially expressed genes, linear modeling of sample groups was performed using ANOVA within Partek Genomic Suites. The magnitude of the response (fold change calculated using the least square mean) and the p-value associated with each probe set and binary comparison was calculated, as well as step-up, adjusted p-value for the purpose of controlling the false discovery rate [81], [82]. Genes were considered to be differentially expressed when the signed fold change was greater than 2 and *P*<0.05.

### Immunohistochemistry

After removing pancreatic, axillary, mesenteric, renal, cervical, and brachial LN as well as spleens, tissue was immediately fixed in 4% formaldehyde, 0.4% picric acid in 1×PBS o/n at 4°C. Tissue was then rinsed 3x for 15 m in PBS and cryoprotected o/n at 4°C in 30% sucrose in 1×PBS. Tissue was stored at −80°C in OCT prior to cryosectioning. Floating sections of colonic tissue from guinea pig gut (obtained in a previous study [75]), were used as positive tissue controls. Tissue was cryosectioned and collected slides and stored at −80°C prior to staining. Tissue was stained with rabbit anti-neuron specific enolase antiserum (Polysciences) diluted at 1∶10,000 followed Cy3-conjugated goat anti-rabbit antibody (Jackson Immunoresearch) at 2.5 µg/ml.

Slides were analyzed with an Olympus AX70 fluorescence photomicroscope. Filter sets for Cy3 were 510 nm-550 nm excitation and 590nm emission. Images were captured with an Optronics Magnafire CCD camera, attached to the Olympus AX70 microscope. Images were cropped in Microsoft PowerPoint with minimal alteration (minor adjustments to brightness and contrast).

### qRT-PCR Validation of Differentially Expressed Genes

WT and H_3_RKO mice were immunized subcutaneously with CFA, followed by i.v. injection of PTX. 24 hours later, draining LN were removed and snap frozen in liquid nitrogen. RNA was extracted using the RNEasy kit (Invitrogen) according to manufacturer’s instructions. cDNA was reverse transcribed using the Taqman Gold RT-PCR kit. qRT-PCR was performed using the DyNAmo Colorflash SYBR green qPCR kit (Thermofisher) and previously described primer sets [71], [83]. *Ywhaz* and *Actb*_were used as reference genes and relative mRNA levels were calculated using the comparative C_T_ method, normalizing to the expression level in WT LN.
